# Development of HTLV-1 Database for data management and datamining HTLV-1 sequences

**DOI:** 10.1186/1742-4690-8-S1-A79

**Published:** 2011-06-06

**Authors:** Thessika H A Araujo, Leandro I Souza-Brito, Pieter Libin, Anne-Mieke Vandamme, Koen Deforche, Bernardo Galvao-Castro, Luiz C Alcantara

**Affiliations:** 1 Advanced Public Health Laboratory, Gonçalo Moniz Research Center, Oswaldo Cruz Foundation, Salvador, Bahia, Brazil; 2 HTLV Center/ Bahia School of Medicine and Public Health/Bahia Foundation for Science Development, Salvador, Bahia, Brazil; 3 Rega Institute for Medical Research, Katholieke Universiteit Leuven, Leuven, Belgium; 4 National Cancer Institute, National Institute of Health, Bethesda, MD, USA

## 

Scientific development had generated a great amount of data that should be stored and managed, in a way, that the users have access to the complete data of interest. The amount of HTLV-1 not related data that had been generated warranties the designing of a database that would be able of put together a greater number of virus data: as epidemiological and environmental aspects, to give support to the inferences about infection, pathogenesis, origin and, principally evolutionary dynamics. All the data was obtained from papers available at GenBank or through contact with the authors. All collected data was organized using the PostgreSQL, and are accessed and managed through the RegaDB: a Viral Data and Analysis Management Environment. We, currently, have 1,972 registered sequences which 23.5% are complete genome and 76.5% are partial genome (Sequences corresponding to LTR, gag, env and pX regions). In relation to clinical status we have collected information for 42.70% of the sequences, and 39.54% of them were obtained from viral isolates of TSP/HAM individuals, while 21.14%, 33.01% and 6.31% of them, were obtained from ATL patients, asymptomatic and other infections, respectively. We also have reports of gender and age in 12.38% and 9.33% of the sequences, respectively. Nowadays, we are dedicating ourselves to the preparation of an interface to permit the access of exterior users, and to the test of the tool utility, and finally to the web publication.

